# Electric Field‐Assisted Uptake of Hexavalent Chromium Ions with In Situ Regeneration of Carbon Monolith Adsorbents

**DOI:** 10.1002/advs.202301419

**Published:** 2023-05-05

**Authors:** Biao Wang, Qi Jiang, Guangxing Yang, Haofan Wang, Hongjuan Wang, Feng Peng, Hao Yu, Jiangnan Huang, Guoyu Zhong, Yonghai Cao

**Affiliations:** ^1^ School of Chemistry and Chemical Engineering Guangdong Provincial Key Lab of Green Chemical Product Technology South China University of Technology 510640 Guangzhou China; ^2^ Guangzhou Key Laboratory for New Energy and Green Catalysis, School of Chemistry and Chemical Engineering Guangzhou University 510006 Guangzhou China; ^3^ College of Chemistry and Chemical Engineering Zhongkai University of Agriculture and Engineering 510225 Guangzhou China; ^4^ School of Chemical Engineering and Energy Technology, Guangdong Provincial Key Laboratory of Distributed Energy Systems Dongguan University of Technology 523808 Dongguan China

**Keywords:** Cr(VI), electrosorption, in situ regeneration, mesoporous carbon monolith

## Abstract

The uptake of hexavalent chromium (Cr(VI)) ions from wastewater is of great significance for environmental remediation and resource utilization. In this study, a self‐designed instrument equipped with an oxidized mesoporous carbon monolith (o‐MCM) as an electro‐adsorbent is developed. o‐MCM with a super hydrophilic surface displayed a high specific surface area (up to 686.5 m^2^ g^−1^). With the assistance of an electric field (0.5 V), the removal capacity of Cr(VI) ions is as high as 126.6 mg g^−1^, much higher than that without an electric field (49.5 mg g^−1^). During this process, no reduction reaction of Cr(VI) to Cr(III) ions is observed. After adsorption, the reverse electrode with 10 V is used to efficiently desorb the ions on the carbon surface. Meanwhile, the in situ regeneration of carbon adsorbents can be obtained even after ten recycles. On this basis, the enrichment of Cr(VI) ions in a special solution is achieved with the assistance of an electric field. This work lays a foundation for the uptake of heavy metal ions from wastewater with the assistance of the electric field.

## Graphical abstract

The efficient uptake of Cr(VI) ions from wastewater with the assistance of an electric field is achieved by using a self‐designed instrument equipped with an oxidized mesoporous carbon monolith as an electro‐adsorbent. The in situ regeneration of carbon adsorbents and the enrichment of Cr(VI) ions in a special solution is obtained with the assistance of an electric field.

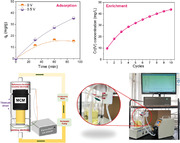

## 1 Introduction

The extraction of heavy metal ions from industrial wastewater is of great challenge in environmental remediation.^[^ ^1^ ^]^ Toxic hexavalent chromium (Cr(VI) ions) is the typical contaminant, which usually causes severe environmental problems.^[^ ^2^ ^]^ More than 1.6 × 10^8^ tons per year of wastewater containing Cr(VI) ions with a concentration of ≈60–1800 mg L^−1^ from the leather processing industry in China are discharged into the environment,^[^ ^2^, ^3^ ^]^ causing the loss of resources, since the price of metallic chromium is ≈10,000 US dollars per ton.^[^ ^3^ ^]^ A lot of efforts are devoted to developing an efficient method for the uptake of chromium from wastewater. Electrosorption is one of the traditional technologies to treat heavy metal wastewater, which has the advantages of low cost, no secondary pollution, and easy regeneration.^[^ ^4^ ^]^ Carbon materials as the adsorbents were used to promote the adsorption process of Cr(VI) ions.^[^ ^5^ ^]^ For example, Liang et al. prepared a novel lignin‐based hierarchical porous carbon (L‐HPC) through a hydrothermally induced assembly and alkali activation strategy. L‐HPC was a multi‐molecular layer adsorbent with an adsorption capacity of 887.8 mg g^−1^ for Cr(VI).^[^ ^6^ ^]^ Zhang et al. prepared polypyrrole/reduced graphene oxide aerogel as an electrode material for the removal of Cr(VI) from the aqueous solution, and the removal rate of Cr(VI) within 30 min was as high as 98.5% (173.56 mg g^−1^) at 25 V.^[^ ^7^ ^]^ Moreover, surface modifications on carbon adsorbents would be helpful in enhancing the performance.^[^ ^8^ ^]^ The introduction of oxygen‐containing functional groups such as carboxyl (—COOH), hydroxyl (—OH), and carbonyl (—C=O) on the carbon surface endows the hydrophilic surface, which is beneficial to improve the transportation of metal ions inside carbons.^[^ ^9^ ^]^ Xie et al. modified the surface of carbon nanotubes as an adsorbent to remove Cr(VI) and found that its removal performance was closely related to the surface chemical properties.^[^ ^10^ ^]^ Bharath et al. prepared a nano‐composite adsorbent with a high mesoporous structure and a large specific surface area by hydrothermal synthesis, and the maximum electrosorption of Cr(VI) under 1.2 V conditions was 24.5 mg g^−1^.^[^ ^11^ ^]^ These studies show that electrosorption technology has a good application for the removal of Cr(VI) ions. However, two essential problems are needed to be solved: i) the reported carbon materials are powder materials, which could not be in situ regenerated and not suitable for practical applications; ii) the uptake of Cr(VI) ions should be achieved alongside with the regeneration of adsorbent, which has not been reported.

Carbon monolith material has a 3D and self‐supporting structure with high specific surface area, high porosity, and good physical/chemical stability,^[^ ^12^ ^]^ which is widely used in the fields of electrochemistry (hydrogen evolution reaction (HER) and oxygen evolution reaction), battery, gas separation and so on.^[^ ^13^ ^]^ For environmental remediation, carbon monolith material has been reported as a unique adsorbent in the removal of organic and inorganic contaminants.^[^ ^14^ ^]^ 3D carbon nanotube sponges were found to be a good adsorbent for the removal of oil and organic substrates.^[^ ^15^ ^]^ The hierarchical carbon aerogel monoliths were utilized for the NaCl desalination with a high removal efficiency.^[^ ^16^ ^]^ Pan et al. reported that graphene aerogel as a conductive and in situ regenerated adsorbent was used for the removal of Cu^2+^ ions with a performance of 68.2 mg g^−1^, close to the theoretical adsorption capacity.^[^ ^4b^ ^]^ Meanwhile, the operation model, such as Flow‐through and Flow‐by models, displayed significantly different performances of the removal of Cr(VI),^[^ ^17^ ^]^ in which typically a higher activity over the Flow‐through model was observed.^[^ ^18^ ^]^ Nevertheless, there are seldom studies reported about the uptake of Cr(VI) ions by using electrosorption technology and carbon monolith materials as adsorbents so far.

Herein, mesoporous carbon material (MCM) as the adsorbent was equipped in a laboratory self‐designed instrument to uptake Cr(VI) ions from the wastewater. An electric field with a low potential (0.5 V) was used in this process to avoid faradaic reactions. The performance of carbon monoliths and adsorption/desorption conditions were comprehensively studied. The mechanism of the electric field‐assisted adsorption technology in the removal of Cr(VI) in an aqueous solution was revealed as well.

## 2 Results and Discussion

### 2.1 Preparation of MCM

**Figure 1:**
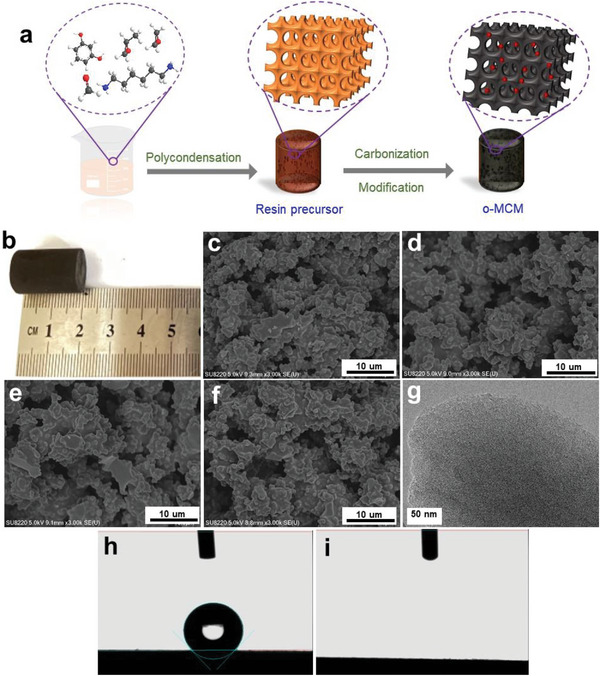
a) Schematic of the preparation process of MCM. b) Optical image of MCM. SEM images of o‐MCMs synthesized at c) 700 °C, d) 800 °C, e) 900 °C, and f) 1000 °C. g) TEM image of o‐MCM900@60. Contact angles of water droplets on h) MCM900 and i) o‐MCM900@60.

In this work, MCM was prepared via a facile method with resorcinol and formaldehyde solution as precursors, and F127 as a soft template, followed by a carbonization process and H_2_O_2_ (3 wt.%) treatment (**Figure** **1**a), which was named o‐MCMX@Y, where X was the pyrolysis temperature, and Y was the modification time. The well‐defined cylindrical shape of o‐MCMs with a size of 14 × 20 mm was observed (Figure 1b). SEM images demonstrated that the holey structure of o‐MCMs was obtained (Figure 1c–f), in which the dense microstructures were typically formed with the variation of the pyrolysis temperature from 700 to 1000 °C, resulting from the polymerization‐induced phase separation.^[^ ^19^ ^]^ In this process, the polymer unit was interconnected to form a 3D network framework, while the liquid‐phase solvent gradually evaporated, leaving porous channels.^[^ ^19^ ^]^ The porous structure offered the high specific surface area (SSA) of o‐MCMs, which increased from 559.9 to 686.5 m^2^ g^−1^ according to the pyrolysis temperature (700–900 °C). Further increasing the temperature to 1000 °C, the SSA of o‐MCM1000 decreased to 359.4 m^2^ g^−1^ (Table S1, Supporting Information), probably due to the decreased thickness of the mesopore wall, reduced micropore volume, and the intensive gasification of carbon at a higher temperature.^[^ ^20^ ^]^ Raman results demonstrated that two broad peaks, as D and G bands, on o‐MCM900@60, were observed, indicating that there were numerous defects on the surface. The value of I_D_/I_G_ gradually increased along with the increase in the pyrolysis temperature (700–1000 °C). In this process, the transformation of an amorphous carbon (a‐C) phase to nanocrystalline graphite would occur, and more edge carbons were produced (Figure S1, Supporting Information).^[^ ^21^ ^]^

The surface oxygen‐containing groups endow carbons with high efficiency in environmental remediations.^[^ ^22^ ^]^ In this study, the process of 3 wt.% H_2_O_2_ treatment would introduce a large number of oxygen‐containing groups on the MCM surface, which enhanced the surface hydrophilicity property based on the contact angle measurements (132°–0°) (Figure 1h,i). Meanwhile, FTIR results clearly showed that the content of (—C=O, 1645 cm^−1^), and —OH groups (3480 cm^−1^) on the surface of the material increased significantly without destroying the surface structure of MCM underwent the H_2_O_2_ treatment (Figure S2, Supporting Information).

### 2.2 Exploring the Adsorption Conditions of Cr(VI)

**Figure 2:**
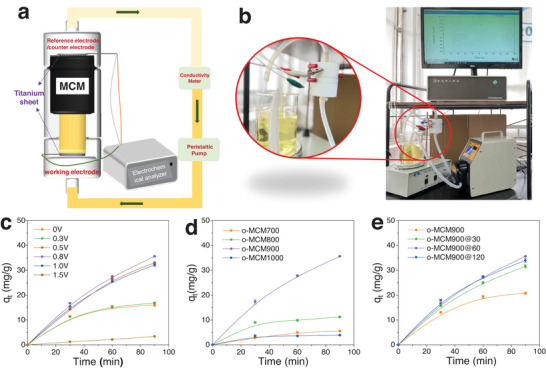
Electrosorption device a) simple diagram, b) physical diagram; The effect of c) voltage, d) pyrolysis temperature, e) H_2_O_2_ modification time on Cr^6+^ removal performance. Conditions: 400 mL, 0.2 g carbon adsorbent, 100 mg L^−1^ Cr(VI), 10 g L^−1^ Na_2_SO_4_, pH = 3 and 5 mL min^−1^, and 0.5 V.

The Cr(VI) removal performance was carried out in the self‐designed equipment by a flow‐through mode, in which the space velocity of the water flow from bottom to top over the carbon monolith was generally controlled at 97.5 h^−1^ with a flow rate of 5 mL min^−1^ (**Figure** **2**a). The references/counter electrodes and carbon monolith meshed together tightly, and a gap between the carbon material and the working electrode was set. The wastewater was returned to the beaker after flowing through the adsorbent material.

The apparent influence of the applied voltage (0–1.5 V) was observed in the removal of Cr(VI) ions with o‐MCM900@60 as the adsorbent. It can be seen that the optimized removal efficiency of 35.67 mg g^−1^ for Cr(VI) ions could be obtained with the assistance of the applied voltage (0.5 V) (Figure 2c). When the applied voltage was below 0.5 V, the removal efficiency would increase alongside the increase of applied voltage, due to the strengthened interactions between the ions and carbons. The higher applied voltage (> 0.5 V) displayed a negative effect on the removal performance, ascribed to the induced Faraday reactions (e.g., ORR and OER reactions).^[^ ^23^ ^]^ These results demonstrate that the Cr(VI) removal over carbon monolith could be efficiently tuned by the applied voltage. Based on this, the performance of o‐MCM900@60 synthesized from different pyrolysis temperatures was investigated. The highest removal efficiency of carbon adsorbents synthesized from 900 °C was achieved (Figure 2d), while the low or higher synthesis temperature displayed comparably decreased activity, which probably could be explained why o‐MCM900@60 had the highest specific surface area (Table S1, Supporting Information).^[^ ^24^ ^]^ The previous publications demonstrated that the oxygenated surface functionality would facilitate the adsorption process. In this work, the treatment of o‐MCM900@60 materials by H_2_O_2_ solution with different concentrations (0–10 wt.%) and treatment times (0–120 min) was conducted (Figure 1a). The optimized MCM materials underwent H_2_O_2_ treatment with 3 wt.% for 60 min and displayed the best adsorption performance (Figure 2e), attributed to the increased amount of hydroxyl group on the carbon surface, which enhanced the interactions between Cr(VI) and the adsorbent.^[^ ^3^, ^25^ ^]^ The aforementioned results show that o‐MCM900@60 was the optimized adsorbent for this study, which could be selected as the typical adsorbent for further investigations.

**Figure 3:**
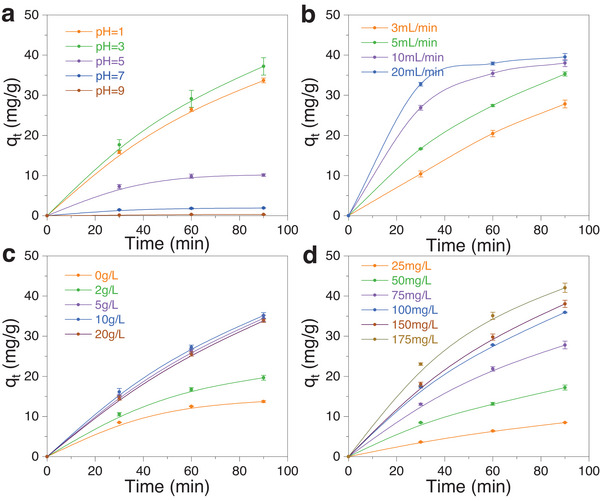
Effects of a) pH, b) flow rate, c) Na_2_SO_4_ concentration, d) solution concentration on Cr(VI) removal performance. Typical conditions: 400 mL solution, 0.2 g carbon adsorbent, 100 mg L^−1^ Cr(VI), 10 g L^−1^ Na_2_SO_4_, pH = 3 and 5 mL min^−1^, 0.5 V.

The value of solution pH plays a significant role in the removal of Cr(VI) (**Figure** **3**a). Since Cr(VI) exists in the aqueous solution in the form of various anions (Cr_2_O_7_^2−^, CrO_4_^2−^, HCr_2_O_7_^−^, and HCrO_4_^−^), a high H^+^ concentration would enhance the oxidizability of Cr(VI) ions, which strengthens the interaction between Cr(VI) ions and carbon materials. The experimental result demonstrated that the adsorption capacity of Cr(VI) on carbon adsorbent in an acidic solution (pH < 7) is higher than that in an alkaline solution (pH > 7), where the best performance was obtained when pH was 3. The conductivity of the solution could dominate the Cr(VI) removal performance.

Figure 3b shows the effect of flow rate on Cr(VI) removal performance. As the flow rate increased from 3 to 20 mL min^−1^, it can be seen that the high flow rate could accelerate the adsorption to reach the adsorption equilibrium, which did not change the equilibrium adsorption capacity of o‐MCMs. The addition of Na_2_SO_4_ can effectively enhance the conductivity of the solution and improve the adsorption performance (Figure 3c). When the concentration of Na_2_SO_4_ was higher than 5 g L^−1^, the adsorption efficiency reached the equilibrium. This also might be caused by the competitive adsorption between Na ions and Cr ions on the carbon surface.

Figure 3d shows the influence of initial Cr(VI) concentrations on the adsorption performance of o‐MCM900@60. When the initial Cr(VI) concentration increased from 25 to 175 mg L^−1^, the adsorption capacity of o‐MCM900@60 increased significantly, indicating that a large number of active sites on the adsorbent surface were applied to adsorb Cr(VI) ions, and the higher Cr(VI) concentration would enhance the collisions between Cr(VI) ions and surface sites, thus speeding up the adsorption process. When the initial concentration of Cr(VI) was higher than 175 mg L^−1^, a tiny increase in adsorption capacity over o‐MCM900@60 was obtained, indicating that the active sites on the surface of the adsorbent were gradually covered by the adsorbed ions. Considering that the presence of the electric field might cause the reduction reaction of Cr(VI) to Cr(III), the gross Cr amount of the solution after the electrosorption was conducted (Figure S3, Supporting Information). It can be found that the concentration of gross Cr ions in the solution was close to that of Cr(VI) during the treatment. It is argued that the change in pH value might affect the chromium oxidation state. Here, we conducted the test with different pH values, showing that a negligible difference between Cr(VI) and total chromium ions was observed (Table S2, Supporting Information). These results indicated that Cr(VI) was mainly adsorbed directly on the surface of o‐MCM900@60, rather than reduced to Cr(III).

**Figure 4:**
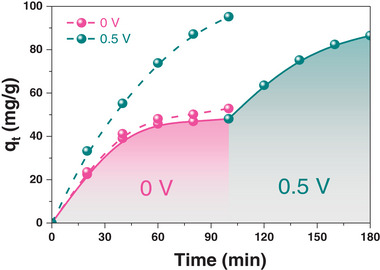
Effect of electric field on Cr(VI) removal performance. Conditions: 400 mL solution, 0.2 g carbon adsorbent, 100 mg L^−1^ Cr(VI), 10 g L^−1^ Na_2_SO_4_, pH = 3 and 5 mL min^−1^.

To visualize the influence of the electric field in the electrosorption process more intuitively, the secondary adsorption with 0.5 V was carried out, where the adsorption equilibrium over material was achieved without an electric field. A significant improvement of the adsorption capacity over o‐MCM900@60 was observed when 0.5 V was applied, which was close to the adsorption performance under the condition of 0.5 V (**Figure** **4**), evidencing that the electric field played a key role in the electrosorption process.

The kinetic models, the pseudo‐first‐order and pseudo‐second‐order models with coefficients of determination (R^2^, 0.9583, and 0.9922, respectively) for the electrosorption process, were also studied (Figure S4, Supporting Information). Chemical adsorption was confirmed to be more favorable to dominate the electrosorption process in this work. The Langmuir and Freundlich adsorption models were used to fit the results (Figure S5 and Table S3, Supporting Information). The experimental results were in good agreement with the Langmuir model (R^2^ is 0.9945), which indicated that a monolayer electrosorption of Cr(VI) on the o‐MCM900@60 electrode was obtained. Under the assistance of an electric field, the maximum Cr(VI) removal capacity (q_max_) of MCM was ≈126.6 mg g^−1^, higher than that of o‐MCM900@60 without an electric field (49.5 mg g^−1^). This result demonstrated a comparable competition to the typical carbon adsorbents in the electrosorption of Cr(VI) removal (Table S4, Supporting Information). Low energy consumption (≈0.5 V) and high adsorption capacity for the treatment of o‐MCM900@60 were obtained, showing a good application prospect for the removal of Cr(VI) in an aqueous solution.

### 2.3 Electrochemical Testing and In Situ Regeneration

**Figure 5:**
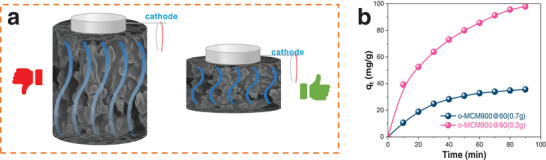
a) Effect of material thickness on electric field; b) effect of material volume on Cr(VI) removal performance. Conditions: 400 mL solution, 0.2 g carbon adsorbent, 100 mg L^−1^ Cr(VI), 10 g L^−1^ Na_2_SO_4_, pH = 3 and 5 mL min^−1^, 0.5 V.

The electrosorption performance of carbon adsorbents highly relies on their conductivity.^[^ ^26^ ^]^ The reverse relationship between the thickness and conductivity of carbon monolith at the end away from the electrode would significantly vary the regeneration activity, which is attributed to the effective distance of the electric field (**Figure** **5**a, Supporting Information). As shown in Figure 5b (Supporting Information), under the same conditions, the adsorption capacity of the thin carbon adsorbent was about 97.95 mg g^−1^, much higher than that of the thick one (35.67 mg g^−1^), which evidenced that the appropriate thickness of o‐MCM900@60 was the essential for fully utilizing the internal or external active sites to remove Cr(VI) ions from aqueous solutions.

The recyclability of adsorption is one of the most important aspects of industrial application. For carbon monolith adsorbents, it is essential to develop a method for in‐situ regeneration after the treatment. Before the test, we have tried several strategies to make the regeneration process easier, more efficient and less chemical reagents, including the effect of the voltage (−2–−10 V), chemical reagents (NaOH and Na_2_SO_4_), and water with room temperature (Figure S9, Supporting Information). It is found that the change in the concentration of Na_2_SO_4_ (2∼10 g/L) could not significantly influence the recyclability of o‐MCM900@60. The increase of applied voltage (−2–−10 V) on o‐MCM900@60 could efficiently increase the recyclability of o‐MCM900@60, but the overall performance decreased inevitably. The temperature also could influence the performance. As we found that when the water at room temperature was used, the lower recyclability of o‐MCM900@60 was observed, due to the that hot water could enhance the desorption of Cr ions from the adsorbent surface. Although the use of NaOH in this process displayed comparable stability, from the aspect of environmental benign in the wastewater treatment, we believe that NaOH itself would be a secondary pollution. Therefore, the process with a relatively high voltage is a considerable choice for this procedure.

**Figure 6:**
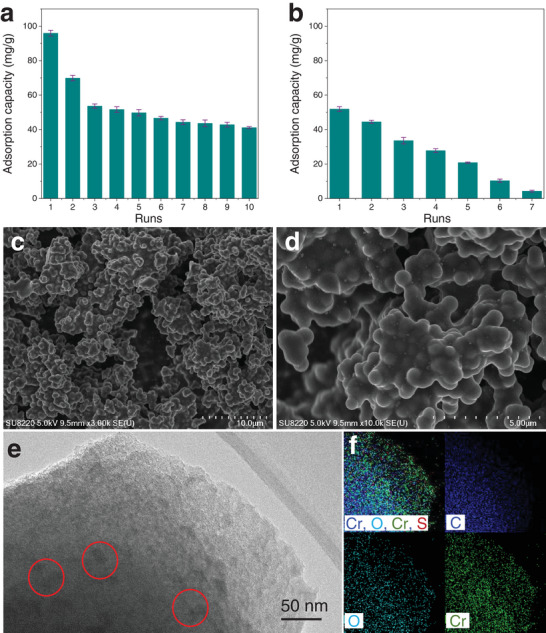
The recyclability of MCM with a) and without electric field b). Adsorption conditions: 400 mL solution, 0.2 g carbon adsorbent, 100 mg L Cr(VI), 10 g L^−1^ Na_2_SO_4_, pH = 3 and 5 mL min^−1^, 0 V (or 0.5 V). desorption conditions: 1000 mL solution, 0.2 g carbon adsorbent, 10 g L^−1^ Na_2_SO_4_, hot water, 0 V (or −10 V). SEM images of o‐MCM900@60 after the electrosorption c,d). TEM e) and elemental mapping f) images of o‐MCM900@60 after the electrosorption.

Here, Cr(VI) removal over o‐MCM900@60 with and without electric field was carried out, in which an in situ regeneration of o‐MCM900@60 by using a negative voltage (−10 V), hot Na_2_SO_4_ solution, and 500 mL of deionized water to desorb Cr(VI) ions on the adsorbent surface. When the adsorption process was performed in the absence of an electric field, the adsorption capacity decreased significantly (**Figure** **6**a). After seven cycles, the adsorption capacity of o‐MCM900@60 was as low as 4.61 mg g^−1^, indicating that there may be a large amount of Cr(VI) ions on the carbon surface, which cannot be desorbed effectively. When an electric field was introduced into the adsorption process, the recyclability of o‐MCM900@60 was improved. The decrease of activity was observed in the first and second cycles, ascribed to the blocked inner micropores of o‐MCM900@60 by the adsorbed ions, which could not be desorbed eventually. This result was also consistent with the declined SSA of the adsorbent after the multiple cycles (Table S1, Supporting Information). After that, high recyclability was observed in the next seven runs (Figure 6a). The removal performance stabilized at about 41.55 mg g^−1^ even at the tenth recycle. These results demonstrated that the presence of the electric field would greatly facilitate the adsorption and desorption of Cr(VI) ions on the carbon surface, which could be helpful for the in situ regeneration of carbon adsorbent. It might be worried that the high potential during desorption probably influenced the chromium oxidation state. Here, we tested the concentration of Cr(VI) and total chromium ions in the solution after the electro‐adsorption and electro‐desorption during the regeneration test. The result showed that no apparent reduction of Cr(VI) ions during the process was obtained (Table S5, Supporting Information). SEM images indicated no obvious change over the structure of o‐MCM900@60 after the adsorption process (Figure 6c). Some particles on the surface of the adsorbent were observed, which might be the Cr salts (Figure 6d), which were further proved by the results of TEM and elemental mapping (Figure 6e,f). The FTIR results showed that the number of hydroxyl groups on the surface of o‐MCM900@60 after adsorption was unchanged (Figure S6, Supporting Information), indicating the high recyclability of the material. The thermogravimetric (TG) results also confirmed that the carbon adsorbent after electro‐desorption still contained some Cr ions inside the material, which could not be completely desorbed and block the inside micropores (Figures S7 and S8, Supporting Information).

### 2.4 Mechanism Study

**Figure 7:**
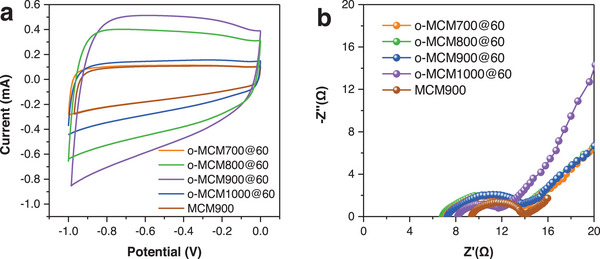
a) Cyclic voltammograms of MCM electrodes at −1.0–0 V with a scan speed of 5 mV s^−1^; b) electrochemical impedance spectroscopy performance images.

It is reported that the conductivity and capacitive property of carbon materials dominate their electrosorption performance.^[^ ^27^ ^]^ A material of high SSA, specific capacitance, and low resistance would be helpful in reducing the diffusion resistance of metal ions into the carbons, which is beneficial for the removal capacity.^[^ ^27b^ ^]^ Therefore, the capacitive performance of the prepared o‐MCMs from 700–1000 °C was evaluated. It can be seen that CV curves of o‐MCMs showed the ideal electric double‐layer capacitance (**Figure** **7**a), indicating excellent reversibility in the experiment. The specific capacitance of o‐MCM900@60 was 178.4 F g^−1^ (Table S6, Supporting Information), significantly higher than that of MCM900 (47.5 F g^−1^), due to the enhanced surface hydrophily of o‐MCM900@60 after the H_2_O_2_ modification (Figure 1h,i), which facilitated the diffusion and accumulation of Cr ions on the carbon surface.^[^ ^28^ ^]^ Figure 7b represented the Nyquist curve of o‐MCMs in the frequency range from 1 MHz to 0.01 Hz. A significantly smaller semicircle radius in the high‐frequency region indicated a low charge transfer impedance over o‐MCM900@60.^[^ ^29^ ^]^ These results demonstrated that the diffusion resistance of o‐MCM900@60 was significantly reduced, and the behavior of the electrode was closer to the ideal electric double layer.^[^ ^30^ ^]^ Meanwhile, the obtained data also agreed with the results measured by IR compensation and multimeter (Table S7, Supporting Information). Herein, the high removal performance of o‐MCM900@60 under the assistance of the electric field could be ascribed to its large capacitance, low resistance, and high conductivity.

**Figure 8:**
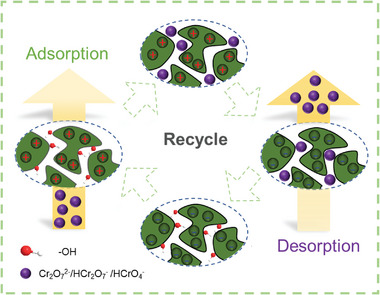
Study on adsorption and electrochemical desorption mechanism in wastewater.

From the aforementioned results, the texture features of o‐MCM900@60 after the electro‐adsorption did not change significantly. And some Cr slats nanoparticles were observed on the surface of the adsorbent, which was also measured by XPS technology (Figure S10, Supporting Information). The high‐resolution deconvolution of Cr XPS was obtained, in which two peaks at 576.7 and 586.6 eV were considered Cr(III) ions, and two peaks at 578.5 and 589.9 eV corresponded to Cr(VI) ions. It can be seen that Cr(VI) was the main existing form in this process. A small amount of Cr(III) ions may be generated due to the reaction between Cr(VI) ions and surface carbon atoms.^[^ ^5a^ ^]^ Combined with the results measured by AAS (Figure S3 and Tables S2 and S5, Supporting Information), Cr(VI) ions in the solution were not converted to Cr(III) ions during the treatment. We speculated that Cr(VI) ions would interact with surface hydroxyl groups or carboxyl groups during the electrosorption process.^[^ ^11^ ^]^ When a reverse electric field was applied, a negative electric field would force the desorption of the complex of Cr(VI), and active surface sites could be regenerated. By this way, we could realize the purpose of desorption (**Figure** **8**).

**Figure 9:**
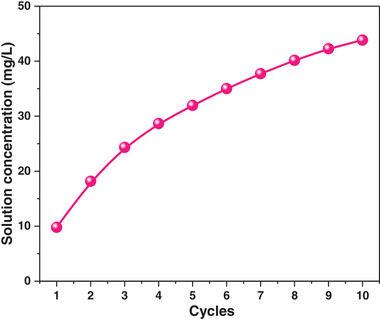
The concentration of Cr(VI) in the Na_2_SO_4_ solution. Conditions (o‐MCM900@60): Adsorption: 400 mL solution, 0.2 g carbon adsorbent, 100 mg L^−1^ Cr(VI), 10 g L^−1^ Na_2_SO_4_, pH = 3 and 5 mL min^−1^, 0.5 V. Desorption: 1000 mL solution, 0.2 g carbon adsorbent, 10 g L^−1^ Na_2_SO_4_ hot water recycled, −10 V.

The uptake of Cr (10 000 dollars per ton) from wastewater is a significant topic for the recycling of environmental resources.^[^ ^31^ ^]^ From the above discussions, carbon monolith adsorbents could be in‐situ regenerated after the efficient desorption of surface ions. Here, we try to utilize this technology to enrich Cr(VI) ions from the wastewater. During this process, a model Cr(VI) solution with an initial Cr(VI) concentration of 100 mg L^−1^ for each adsorption‐desorption process was used. After the adsorption, the Cr(VI) ions on the carbon surface would be electro‐desorbed into a consistent Na_2_SO_4_ solution with 10 mg L^−1^ to enrich the Cr(VI) ions. As shown in **Figure** **9**, with the increase of adsorption‐desorption cycles, the concentration of Cr(VI) ions gradually increased to 45 mg L^−1^ after ten cycles. These results demonstrated that the uptake of Cr(VI) ions with the assistance of an electric field displayed a good potential for the treatment of wastewater from electroplating, leather tanning, and steel manufacturing.^[^ ^32^ ^]^

## 3 Conclusion

In summary, the efficient uptake of Cr(VI) ions from wastewater with the assistance of an electric field was achieved by using self‐designed equipment. o‐MCM900@60 with a high BET SSA (686.5 m^2^ g^−1^) as the typical adsorbent was selected. The electric field with a voltage of 0.5 V for the adsorption was utilized. The research results demonstrated that the removal capacity of Cr(VI) was 126.6 mg g^−1^, which was much better than the system without an electric field (49.5 mg g^−1^). The high electrical conductivity and the hydrophilic surface of o‐MCM900@60 were of great significance to the improvement of the adsorption capacity. The high recyclability of this technology endowed an efficient enrichment of Cr(VI) ions from wastewater, which could be enriched in another solution. This developed technology of environmental remediation has broad application prospects for the uptake of heavy metal ions from environmental wastewater.

## Conflict of Interest

The authors declare no conflict of interest.

## Supporting information

Supporting Information

## Data Availability

The data that support the findings of this study are available from the corresponding author upon reasonable request.
