# Supplementary material for: Electric Field‐Assisted Uptake of Hexavalent Chromium Ions with In Situ Regeneration of Carbon Monolith Adsorbents
Source: Adv Sci (Weinh). 2023 May 5;10(21):2301419. doi: 10.1002/advs.202301419 (PMC10375139; doi:10.1002/advs.202301419)
Supplement: Supplementary file 1 — Supporting Information [file ADVS-10-2301419-s001.pdf]

ADVANCED SCIENCE
Open Access

Supporting Information

for *Adv. Sci.*, DOI 10.1002/advs.202301419

Electric Field-Assisted Uptake of Hexavalent Chromium Ions with In Situ Regeneration of Carbon Monolith Adsorbents

Biao Wang, Qi Jiang, Guangxing Yang, Haofan Wang, Hongjuan Wang, Feng Peng, Hao Yu, Jiangnan Huang, Guoyu Zhong and Yonghai Cao**

Supporting information

Electric Field-Assisted Uptake and Enrichment of Hexavalent Chromium Ions with In-Situ Regeneration Carbon Monolith Adsorbents

Biao Wang[1], Qi Jiang[1], Guangxing Yang[1], Haofan Wang[1], Hongjuan Wang[1], Feng Peng[3], Hao Yu[1], Jiangnan Huang[1, 2*], Guoyu Zhong[4], Yonghai Cao[1*]

[1]*School of Chemistry and Chemical Engineering, Guangdong Provincial Key Lab of Green Chemical Product Technology, South China University of Technology, Guangzhou, 510640, China*

[2]*College of Chemistry and Chemical Engineering, Zhongkai University of Agriculture and Engineering, Guangzhou 510225, China*

[3]*Guangzhou Key Laboratory for New Energy and Green Catalysis, School of Chemistry and Chemical Engineering, Guangzhou University, Guangzhou 510006, China*

[4]*School of Chemical Engineering and Energy Technology, Guangdong Provincial Key Laboratory of Distributed Energy Systems, Dongguan University of Technology, Dongguan, 523808 China*

*Corresponding author:

E-mail: meyhcao@scut.edu.cn (Y. Cao)

cejnhuang@scut.edu.cn (J. Huang)

Experimental section

1. Materials and chemical reagents

Resorcinol, formaldehyde, anhydrous sodium sulfate, and anhydrous ethanol were purchased from Tianjin Damao Chemical Reagent Factory. Block polyether F-127 was purchased from Shanghai Yuanye Biotechnology Co., Ltd. Potassium dichromate ($K_2Cr_2O_7$) was purchased from Sahn Chemical Technology (Shanghai) Co., Ltd. All reagents were directly used without a further treatment.

2. Synthesis of o-MCM

Using resorcinol and formaldehyde as carbon material precursors, a three-dimensional carbon monolith was synthesized by a self-assembly high-temperature carbonization method.[1] Typically, 45 g of resorcinol and 18.75 g of block polyether F127 were dissolved in a solvent mixture of 152 mL of absolute ethanol and 120 mL of deionized water under magnetic stirring at 25 °C, followed by the addition of 1.56 g of 1,6 hexanediol. The amine was added to the above solution and stirred for about 10 min, then 61.5 mL of formaldehyde solution was quickly added to the mixed solution and stirred for 20 min. The obtained white homogeneous emulsion was then transferred to an oven at 50 °C for 24 h, and then continued to be cured at 70 °C for 24 h and 100 °C for 12 h to obtain the solid reactant phenolic resin. The monolithic mesoporous carbon material (MCM) was obtained by pyrolyzing the phenolic resin at 700, 800, 900 and 1000 °C for 2 h at a heating rate of 2 °C/min in an Ar atmosphere, respectively. Finally, the MCM material was immersed in 100 mL of 3%wt H_2O_2 solution for a certain time by ultrasonic immersion to improve its hydrophilicity. The obtained MCM materials could be o-MCMT@X, T and X are noted as the pyrolysis temperature and

ultrasonic time, respectively.

3. Characterization

The surface morphology and microstructure of the prepared samples were characterized by field emission scanning electron microscope (SEM, Zeiss Merlin) at 5 kV. Transmission electron microscopy (TEM) was taken in a JEOL JEM-2100F microscope operating at 200 kV. The specific surface area (BET) of the samples was measured on an ASAP 2010 analyzer by N_2 adsorption. Raman spectra were obtained from a LabRAM Arram Micro Raman spectrometer with an excitation wavelength of 633 nm. X-ray photoelectron spectroscopy (XPS) was performed on a quito-axis ultrasonography (DLD) spectrometer equipped with an aluminum X-ray source, and the binding energy was referenced to the C1s peak at 284.6 eV. Fourier transform infrared spectroscopy (FTIR) was used to analyze the surface functional groups at wavelengths of 1500-4000 cm^{-1} by Nicolet IS50 infrared microscope. Thermogravimetric analysis (TG) was performed by heating the sample to 1000°C at a heating rate of 10°C/min in an air atmosphere by STA449C. The resistance of the material is measured by VICTOR VC890C+ multimeter.

4. Electrochemical testing

Cyclic voltammetry (CV) and electrochemical impedance (EIS) measurements were performed using an electrochemical analyzer (CHI660E, CH Instruments, USA). In the CV test, platinum and silver/silver chloride electrodes were used as counter and reference electrodes, respectively. Conductive carbon paper was used as the working electrode by coating MCM, naphthol solution and ethanol solution. All CV measurements were performed with 0.1 M $KHCO_3$ solution. The electrolyte was degassed with purified argon before CV

measurements, and argon was passed through the solution during all measurements, The scan rate was set to 5 mV/s. In the EIS test and IR compensation experiment, platinum and silver/silver chloride electrodes were used as counter and reference electrodes, respectively. The MCM was ground and used as the working electrode. All measurements were performed with 0.1 M Na_2SO_4 solution. The electrolyte was degassed with purified argon gas prior to measurement, and argon gas was continuously bubbled into the solution during the measurement.

5. Electro-sorption/desorption performance test

Electro-adsorption: In this experiment, potassium dichromate was used as the model pollutant, MCM was used as the adsorbent, and a self-designed equipment was used as the electrosorption device with a two-electrode system (**Figure 1**). We set the H_2O_2-modified MCM adsorbent as the working electrode, and 400 mL potassium dichromate solution was adsorbed by MCM (~0.7 g) through a peristaltic pump under the continuous stirring. In this work, the carbonization temperature (700~1000 ℃), potassium dichromate solution concentration (25~200 mg/L), applied voltage value (0~1.5 V), pH (1~13), electrical conductivity of MCM were studied respectively. Effects of medium concentration (0~20 g/L) and H_2O_2 modification time on removal of chromium ions were also tested. The concentration changes of hexavalent chromium and total chromium ions were determined by the ultraviolet spectrophotometer and flame atomic absorption photometer (AAS).

Electrode regeneration: 1 L of 90 °C sodium sulfate solution (10 g/L) and 500 mL of deionized water at a flow rate of 20 mL/min were forcedly passed through the adsorbed o-MCM900@60 with a negative voltage (10 V) for the electrode regeneration.

Enrichment of Cr

Cr(VI) enrichment: This process is similar to the regeneration experiment. 1 L of 90 °C sodium sulfate solution (10 g/L) with a flow rate of 20 mL/min in the desorption process is used repeatedly. The adsorbed chromium ions are enriched into the same sodium sulfate solution.

6. Isotherms and kinetic study

In order to describe the electroadsorption kinetics of Cr(VI) on MCM adsorbent, pseudo-first-order and pseudo-second-order kinetic models were used to fit the experimental data.[2] The linear forms of these two models can be expressed as follows,

Pseudo-first-order:

$$\log(q_e - q_t) = log q_e - \frac{k_1}{2.303} t \tag{S1}$$

Pseudo-second-order:

$$\frac{t}{q_t} = \frac{1}{k_2 q_e^2} + \frac{t}{q_e} \tag{S2}$$

where q_e and q_t are the electrosorption capacities at equilibrium conditions and at time t, respectively, t is the total electrosorption time, and k_1 and k_2 are the rate constants of the pseudo-first- and pseudo-second-order equations, respectively.

The adsorption isotherms of the adsorbents were fitted by Langmuir and Freundlich models with 0 V and 0.5 V applied, respectively [3].

Langmuir model：

$$\frac{C_e}{q_e} = \frac{1}{b q_{max}} + \frac{C_e}{q_{max}} \tag{S3}$$

Freundlich model:

$$Log qe = Log k_f + \frac{1}{n} Log C_e \tag{S4}$$

where C_e (mg/L) is the initial Cr^{6+} concentration, q_e (mg/g) is the amount of Cr^{6+} adsorbed on the adsorbent under equilibrium conditions, q_{max} (mg/g) is the maximum adsorption amount of the adsorbent, *b* (L/mg), k_f and *n* are constants in the models.

Table and Figure Captions

Table S1 Texture properties of o-MCMs. 8

Table S2 The concentration of chromium in the solution before and after electro-adsorption with different pH value........ 9

Table S3 Parameters of Langmuir and Freundlich models over o-MCM900@60 at 0 V and 0.5 V........ 9

Table S4 Performance comparison for the removal of Cr(VI) over different adsorbents. 10

Table S5 The concentration of chromium in the solution before and after the stability test.[a] 11

Table S6 Specific capacitances of different o-MCMs. 12

Table S7 Measured resistances of o-MCMs with IR compensation and multimeter........ 13

Figure S1 Raman spectral of o-MCMs synthesized from the different pyrolysis temperatures. 14

Figure S2 FTIR results of MCMs before and after H_2O_2 modification. 15

Figure S3 Effects of (a) solution concentration, (b) voltage on total chromium removal performance. Conditions: 400 mL, 0.2 g carbon adsorbent, 100 mg/L Cr(VI), 10 g/L Na_2SO_4, pH=3, 5 mL/min, 0.5 V........ 16

Figure S4 The adsorption kinetics of Cr(VI) by o-MCM900@60 were fitted by (a) pseudo-first-order model and (b) pseudo-second-order model........ 17

Figure S5 18

Figure S6 FTIR results of o-MCM900@60 before and after electro-adsorption........ 19

Figure S7 TG test of o-MCM900@60 before and after the electro-adsorption. 20

Figure S8 TG test of o-MCM900@60 after 10 runs. 21

Figure S9 Effects of (a) Na_2SO_4 concentration, (b) voltage, (c) temperature, (d) solution medium on Cr(VI) regeneration performance. Conditions: 1000 mL, 0.2 g carbon adsorbent, 10 g/L Na_2SO_4 (or 4 g/L NaOH), -10 V, hot or cold water, 20 mL/min........ 22

Figure S10 (a) XPS survey and (b) the high-resolution deconvolution of Cr 2p after the electro-adsorption of o-MCM900@60........ 23

Table S1 Texture properties of o-MCMs.

	Average pore size (nm)	Pore volume (cm^3/g)	SSA (m^2/g)
o-MCM700@60	3.59	0.358	559.9
o-MCM800@60	3.70	0.400	572.6
o-MCM1000@60	3.24	0.238	359.4
MCM900	3.95	0.385	604.5
o-MCM900@30	3.81	0.402	604.3
o-MCM900@60	2.57	0.369	686.5
o-MCM900@120	2.65	0.312	590.0
o-MCM900@60 absorbed	3.12	0.309	527.4
o-MCM900@60 after 10 times cycles at 10 V	3.88	0.333	483.3
o-MCM900@60 after 7 times cycles at 0 V	4.01	0.355	476.7

Table S2 The concentration of chromium in the solution before and after electro-adsorption with different pH value

	pH=1		pH=3		pH=5		pH=7		pH=9	
	before	after	before	after	before	after	before	after	before	after
Cr(VI) (mg/L)	100.1±0.05	54.2±0.19	98.2±0.11	50.1±0.3	99.7±0.11	86.3±0.11	99.7±0.17	95.9±0.22	101.5±0.15	100.9±0.02
Total Cr (mg/L)	100.3±0.07	58.9±0.22	97.9±0.09	50.3±0.09	99.7±0.07	88.3±0.22	100.6±0.11	96.3±0.16	102.1±0.17	101.4±0.09

[a] Adsorption conditions: 400 mL solution, 0.2 g carbon adsorbent, 5 g/L Na_2SO_4, pH=3, 5 mL/min, 0.5 V.

Table S3 Parameters of Langmuir and Freundlich models over o-MCM900@60 at 0 V and 0.5 V.

	Langmuir			Freundlich		
	q_{max} (mg/g)	b	r^2	n	K_f	r^2
0 V	49.5	0.36	0.9994	14.9	35.27	0.949
0.5 V	126.6	0.1	0.9991	4.02	38.71	0.961

Table S4 Performance comparison for the removal of Cr(VI) over different adsorbents.

Adsorbent	pH	SSA (m^2/g)	Voltage	Initial concentration （mg/L）	Electro-adsorption capacity (mg/g)	Ref.
Activated carbon	7.2	—	1.2	10	0.9	[4]
		—		100	3.7	
porous carbon	7.0	952	1.0	30	3.3	[5]
G/AgCS aerogel	2.0	61	0.0	10	12.7	[6]
Polyaniline @XAD-4	7.0	747.6	0.0	10	9.2	[7]
Fe_3O_4-BAC	3.0	—	0.0	150	27.9	[8]
SWCNTs@SSNE	4.0	380	1.0	6.38	12.5	[9]
			2.5	6.38	160.4	
o-MCM900@60	3.0	686.5	0.5	100	126.6	This work

Table S5 The concentration of chromium in the solution before and after the stability test.[a]

	Electro-adsorption at 1st run	Electro-desorption at 1st run	Electro-adsorption at 2nd run	Electro-desorption at 2nd run	Electro-adsorption at 3rd run	Electro-desorption at 3rd run
Cr(VI) (mg/L)	52.2±0.06	15.59±0.02	67.2±0.17	12.81±.005	72.85±0.06	10.23±0.02
Total Cr (mg/L)	53.42±0.27	15.82±0.07	66.85±0.27	13.15±0.14	72.33±0.14	10.41±0.14

[a] Adsorption conditions: 400 mL solution, 0.2 g carbon adsorbent, 100 mg/L Cr(VI), 5 g/L Na_2SO_4, pH=3, 20 mL/min, 0.5 V. Desorption conditions: 1000 mL solution, 0.2 g carbon adsorbent, 10 g/L Na_2SO_4, hot water, -10 V.

Table S6 Specific capacitances of different o-MCMs.

	Area（AV）	Scan rate (mV/s)	Potential window (V)	Mass (g)	Specific capacitance（F/g）
o-MCM700@60	2.56E-04	5	1	0.0005	51.3
o-MCM800@60	7.28E-04	5	1	0.0005	145.6
o-MCM900@60	8.92E-04	5	1	0.0005	178.4
o-MCM1000@60	3.78E-04	5	1	0.0005	75.5
MCM900	2.38E-04	5	1	0.0005	47.5

Table S7 Measured resistances of o-MCMs with IR compensation and multimeter.

	IR compensation（Ω）	Multimeter (Ω)
o-MCM700@60	12.3	11.8
o-MCM800@60	12.4	12.9
o-MCM900@60	12.7	14.7
o-MCM1000@60	15.0	18.9
MCM900	18.6	26.2

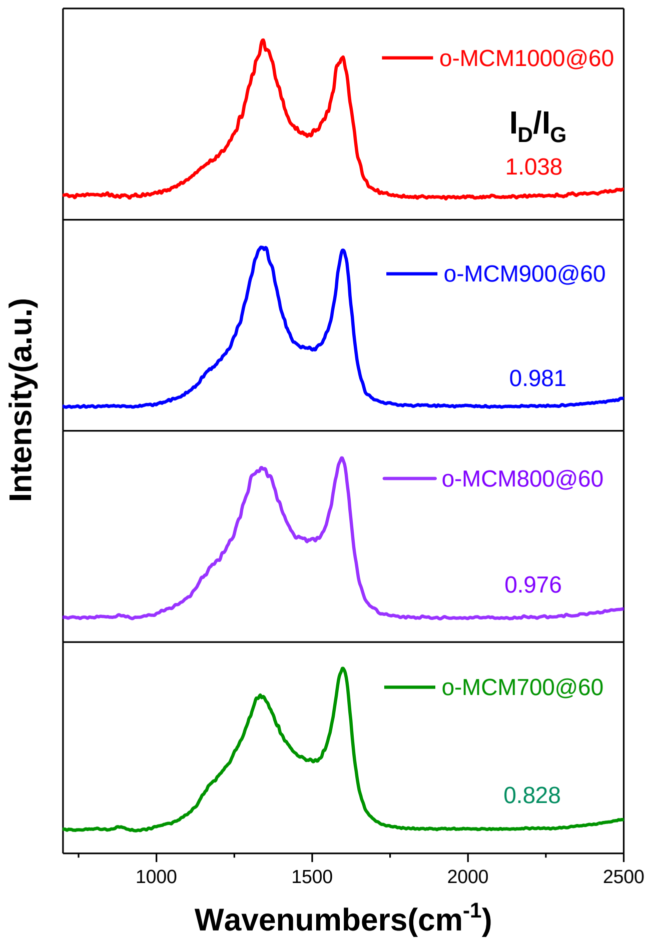

Figure S1 Raman spectral of o-MCMs synthesized from the different pyrolysis temperatures.

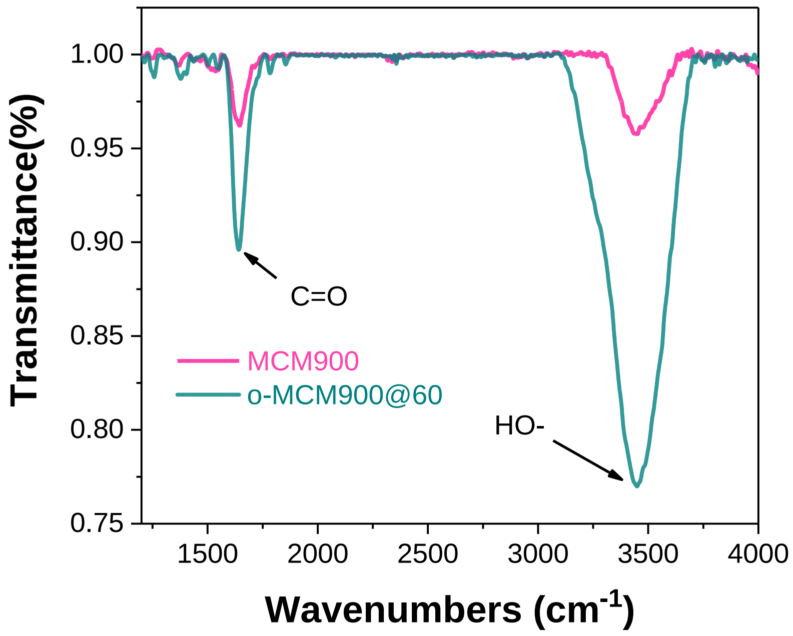

Figure S2 FTIR results of MCMs before and after H_2O_2 modification.

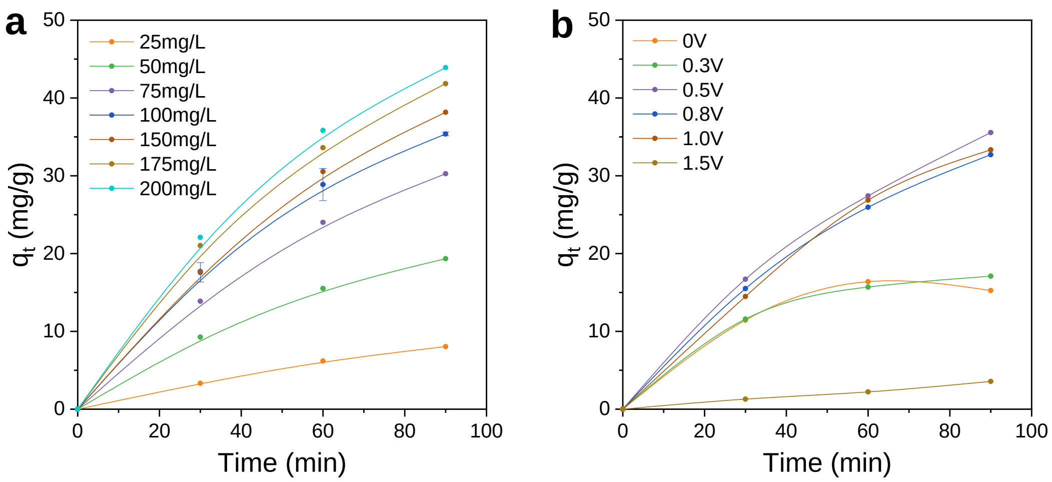

Figure S3 Effects of (a) solution concentration, (b) voltage on total chromium removal performance. Conditions: 400 mL, 0.2 g carbon adsorbent, 100 mg/L Cr(VI), 10 g/L Na_2SO_4, pH=3, 5 mL/min, 0.5 V.

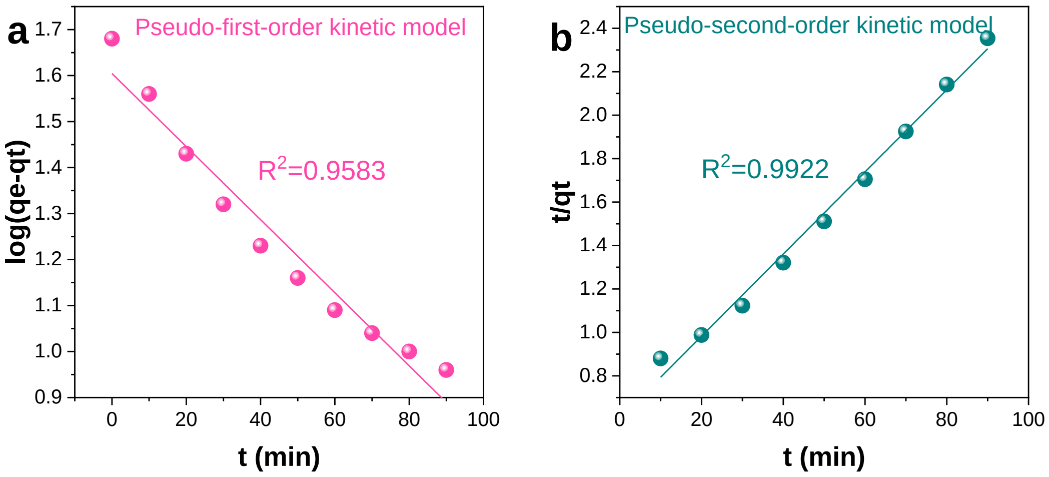

Figure S4 The adsorption kinetics of Cr(VI) by o-MCM900@60 were fitted by (a) pseudo-first-order model and (b) pseudo-second-order model.

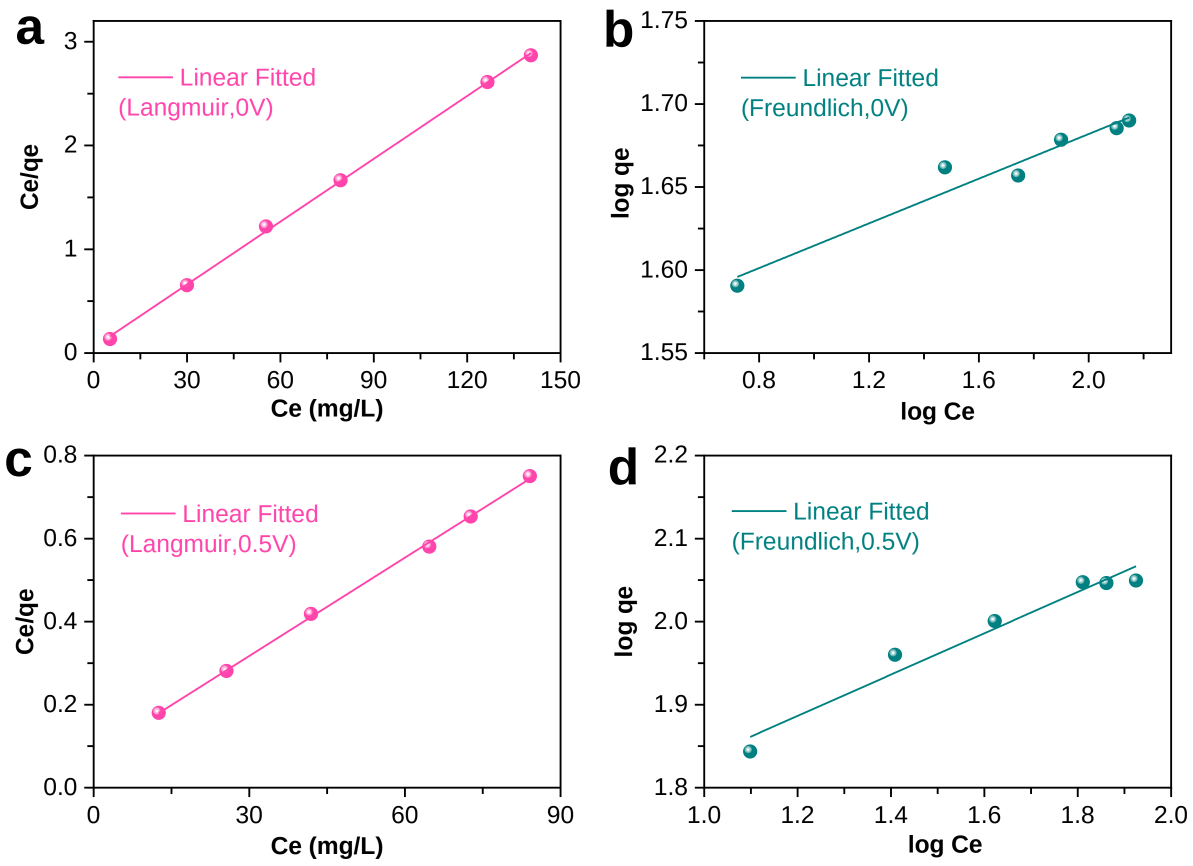

Figure S5 Cr(VI) adsorption isotherms fitted by Langmuir (a, c) and Freundlich (b, d) models for o-MCM900@60 at 0 V (a, b) and 0.5 V (c, d).

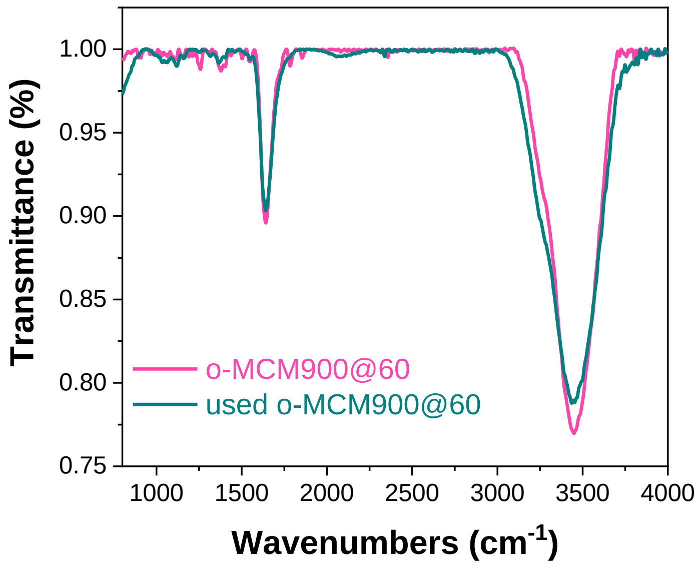

Figure S6 FTIR results of o-MCM900@60 before and after electro-adsorption.

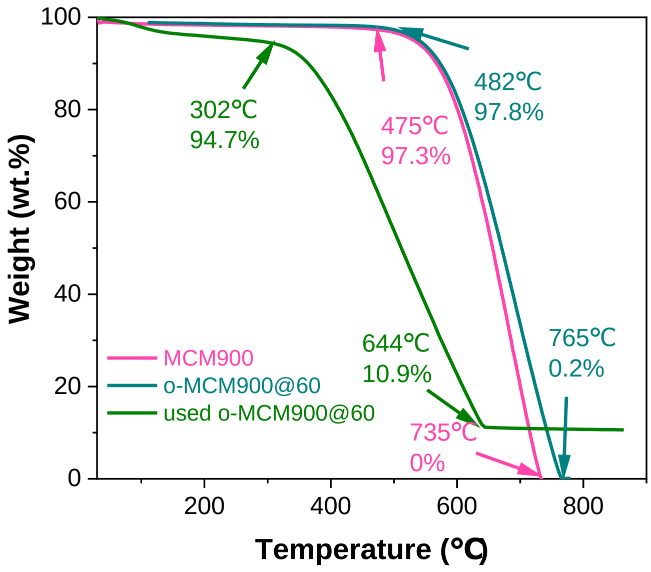

Figure S7 TG test of o-MCM900@60 before and after the electro-adsorption.

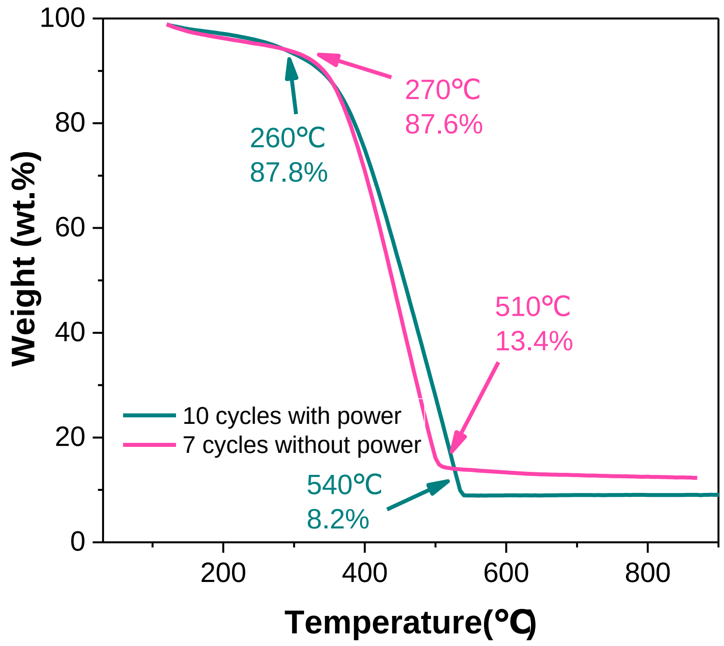

Figure S8 TG test of o-MCM900@60 after 10 runs.

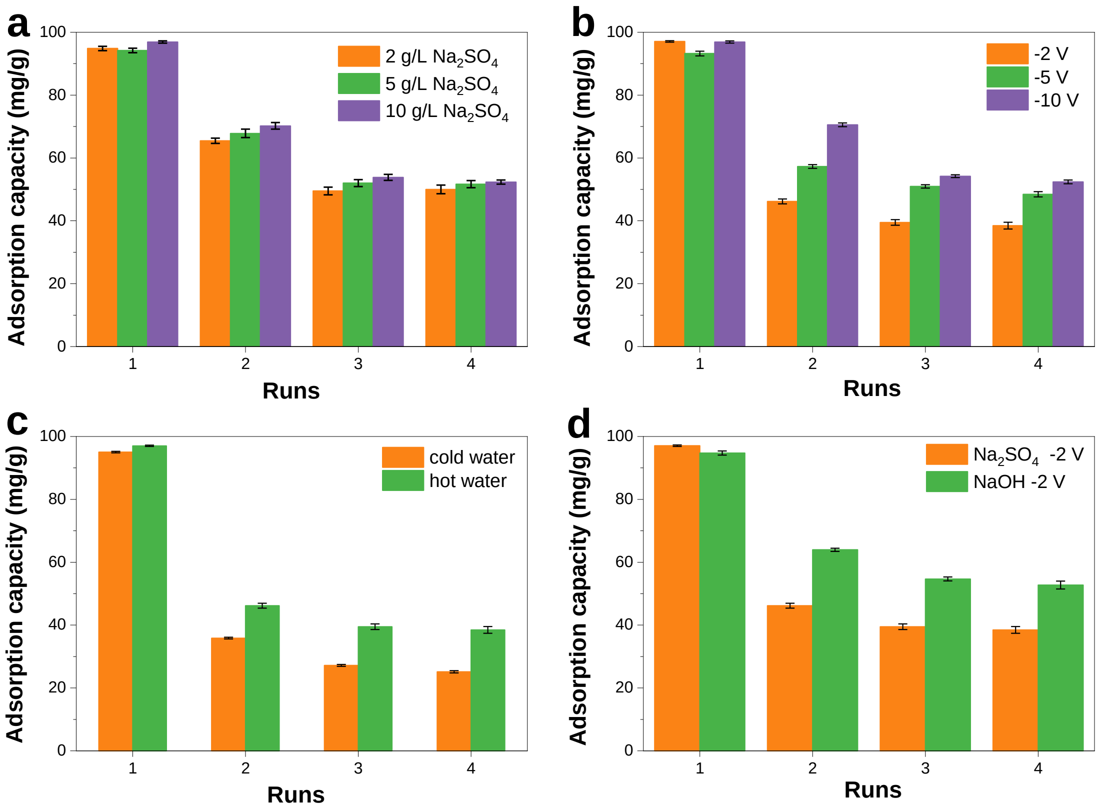

Figure S9 Effects of (a) Na_2SO_4 concentration, (b) voltage, (c) temperature, (d) solution medium on Cr(VI) regeneration performance. Typical conditions: 1000 mL solution, 0.2 g carbon adsorbent, 10 g/L Na_2SO_4 (or 4 g/L NaOH), -10 V, hot (cold water, if applicable), 20 mL/min.

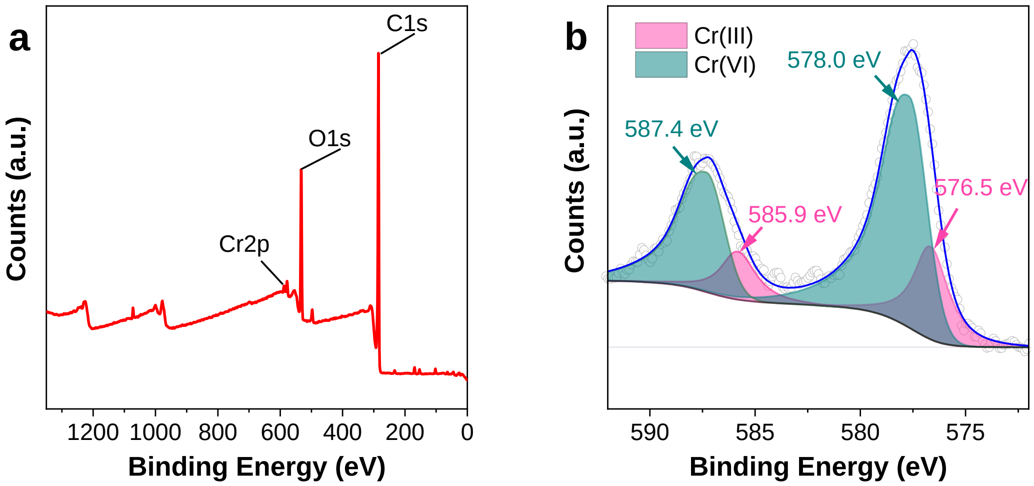

Figure S10 (a) XPS survey and (b) the high-resolution deconvolution of Cr 2p after the electro-adsorption of o-MCM900@60.

References

[1] K. Wang, Y. Zeng, W. Lin, X. Yang, Y. Cao, H. Wang, F. Peng, H. Yu, *Carbon* **2020**, *167*, 709.

[2] a) M. S. Gaikwad, C. Balomajumder, A. K. Tiwari, *Chemosphere* **2020**, *254*, 126781; b) S. Zhang, H. Chen, S. Zhang, C. Kai, M. Jiang, Q. Wang, Z. Zhou, *Cellulose* **2019**, *26*, 3437.

[3] a) A. Buffa, D. Mandler, *Chem. Eng. J.* **2019**, *359*, 130; b) X. Zhang, B. Ren, X. Wu, X. Yan, Y. Sun, H. Gao, F. Qu, *ACS Omega* **2021**, *6*, 25389.

[4] M. S. Gaikwad, C. Balomajumder, *Sep. Purif. Technol.* **2017**, *186*, 272.

[5] X. F. Zhang, B. Wang, J. Yu, X. N. Wu, Y. H. Zang, H. C. Gao, P. C. Su, S. Q. Hao, *RSC Adv.* **2018**, *8*, 1159.

[6] S. P. Dubey, A. D. Dwivedi, I.-C. Kim, M. Sillanpaa, Y.-N. Kwon, C. Lee, *Chem. Eng. J.* **2014**, *244*, 160.

[7] J. Ding, Y. Pan, L. Li, H. Liu, Q. Zhang, G. Gao, B. Pan, *Chem. Eng. J.* **2020**, *384*.

[8] D. Prabu, P. S. Kumar, B. S. Rathi, S. Sathish, K. V. Anand, J. A. Kumar, O. B. Mohammed, P. Silambarasan, *Environ. Res.* **2022**, *203*, 111813.

[9] Y. X. Liu, D. X. Yuan, J. M. Yan, Q. L. Li, T. Ouyang, *J. Hazard. Mater.* **2011**, *186*, 473.
